# Association between cancer and cardiovascular disease risk: a cross-sectional study of 241,064 individuals

**DOI:** 10.3389/fonc.2026.1734601

**Published:** 2026-02-04

**Authors:** Lianmin Zhu, Wenqing Zhou, Yukang Yang, Huan Rao, Yizhi Liu, Yunwei Rao

**Affiliations:** 1 Department of Respiratory and Critical Care Medicine, The First Affiliated Hospital of Gannan Medical University, Ganzhou, China; 2 Department of Emergency, The People’s Hospital of Wanzai County, Yichun, China; 3 Graduate School, Gannan Medical University, Ganzhou, China; 4 Department of Internal Medicine, Zhangshu People’s Hospital, Yichun, China; 5 The First Clinical Medical College, Gannan Medical University, Ganzhou, China

**Keywords:** behavioral risk factor surveillance system, cancer survivors, cardiovascular disease, cross-sectional study, risk factors

## Abstract

**Background:**

Advances in cancer therapy have improved survival, yielding a larger population of cancer survivors, with cardiovascular disease (CVD) becoming a major chronic health issue among them. However, the association between cancer and CVD risk remains unclear.

**Methods:**

This study conducted a cross-sectional analysis utilizing 2023 United States Behavioral Risk Factor Surveillance System (BRFSS) data. Propensity score matching (PSM) balanced the observed covariates between the cancer and non-cancer groups. Univariate and multivariate logistic regression were employed to examine the relationship between cancer history and CVD. Subgroup analyses evaluated this association across different populations. Finally, univariate and multivariate logistic regression identified CVD risk factors among cancer survivors.

**Results:**

Before PSM, CVD prevalence was 20.7% in cancer patients, significantly higher than 10.7% in non-cancer individuals. Multivariate logistic regression revealed that cancer remains positively associated with CVD risk even after adjusting covariates (OR = 1.16, 95% CI: 1.12-1.20, P < 0.001). Analysis after 1:2 PSM further validated this association: The prevalence of CVD was higher in the cancer group than in the matched control group (20.7% vs. 18.2%; OR = 1.14, 95% CI: 1.10–1.18, P < 0.001). Except for diabetes and chronic kidney disease (CKD) subgroups, most subgroups of cancer are positively associated with CVD risk (OR > 1, P < 0.05). Among cancer survivors, advanced age, comorbidities (hypertension, diabetes, CKD), depression, and smoking were significantly positively associated with CVD risk, while female sex, aerobic physical activity, and higher income were significantly negatively associated with cardiovascular disease risk.

**Conclusion:**

There is a significant positive correlation between cancer and CVD risk, indicating that CVD risk assessment and prevention efforts for cancer survivors should be strengthened.

## Introduction

Cardiovascular disease (CVD) constitutes the primary cause of death worldwide. Its high incidence, mortality, and complex pathological subtypes, impose a significant burden on healthcare services and on society (1, 2). Global Burden of Disease 2022 assessment reveals notable regional variation in age-standardized CVD mortality (73.6 per 100,000 in high-income Asia Pacific to 432.3 per 100,000 in Eastern Europe). Despite a 34.9% global decline in age-standardized CVD mortality over the past three decades due to therapeutic advances (3), the absolute number of CVD deaths has risen substantially due to population growth, placing immense pressure on healthcare systems (4). Preventing CVD is therefore critically important for global public health.

Cancer is the second major cause of death in the world, imposing a substantial and expanding global burden. In 2022, about 20,000,000 new cancer cases and 9,700,000 cancer deaths worldwide (5). Advances in screening methods and treatment technologies have reduced mortality, extended survival, and contributed to the continued increase in the number of cancer survivors worldwide (6, 7). This extended survival has shifted focus to managing non-cancer-related comorbidities, particularly given the high risk of CVD mortality in cancer patients (8). Cancer and CVD, two major causes of death, have long been primary focal points of health research, with previous studies reporting shared pathophysiological mechanisms (9, 10). However, cancer remains a subject of debate regarding its association with an increased risk of CVD (11).

It is plausible that cancer survivors experience an elevated risk of CVD owing to shared risk factors, including advanced age, hyperglycemia, hypertension, dyslipidemia, and obesity (12). Furthermore, cancer therapies, including radiotherapy, anthracycline chemotherapy, immunotherapy, can induce cardiotoxicity by directly damaging vascular endothelium or cardiomyocytes, exacerbating cardiovascular injury (13). Nevertheless, other studies have found lower CVD risk in specific cancer survivor groups, such as gastric cancer survivors or those with localized prostate cancer (14, 15). These conflicting findings challenge the consistency of the cancer-survivorship–CVD risk association. It is necessary to conduct a thorough, systematic study to ascertain whether cancer survivors indeed face a higher CVD risk.

Given the complex and debated interplay between cancer and CVD, this study utilized datasets of the US Behavioral Risk Factor Surveillance System (BRFSS). Propensity score matching (PSM) was applied to address potential confounding factors. This study addresses three research questions: (1) Is a history of cancer independently associated with CVD risk? (2) Does this association exhibit heterogeneity across different demographic and clinical subgroups? (3) Which groups of cancer survivors face a high risk of CVD? and which modifiable factors might prevent it? The findings provide crucial evidence for cardiovascular risk stratification and targeted prevention in cancer survivors.

## Methods

### Data source

This study utilized data from the BRFSS database, a nationwide health behavior and chronic disease surveillance system coordinated by the US CDC. Data were collected via telephone surveys and are widely used for public health decision-making and research (16). This study utilized data from 433,323 respondents surveyed in 2023, including health-related behaviors, chronic health conditions, and utilization of preventive services. As BRFSS data are publicly available and de-identified (https://www.cdc.gov/brfss), no Institutional Review Board approval is required.

### Data inclusion and exclusion criteria

**Figure 1:**
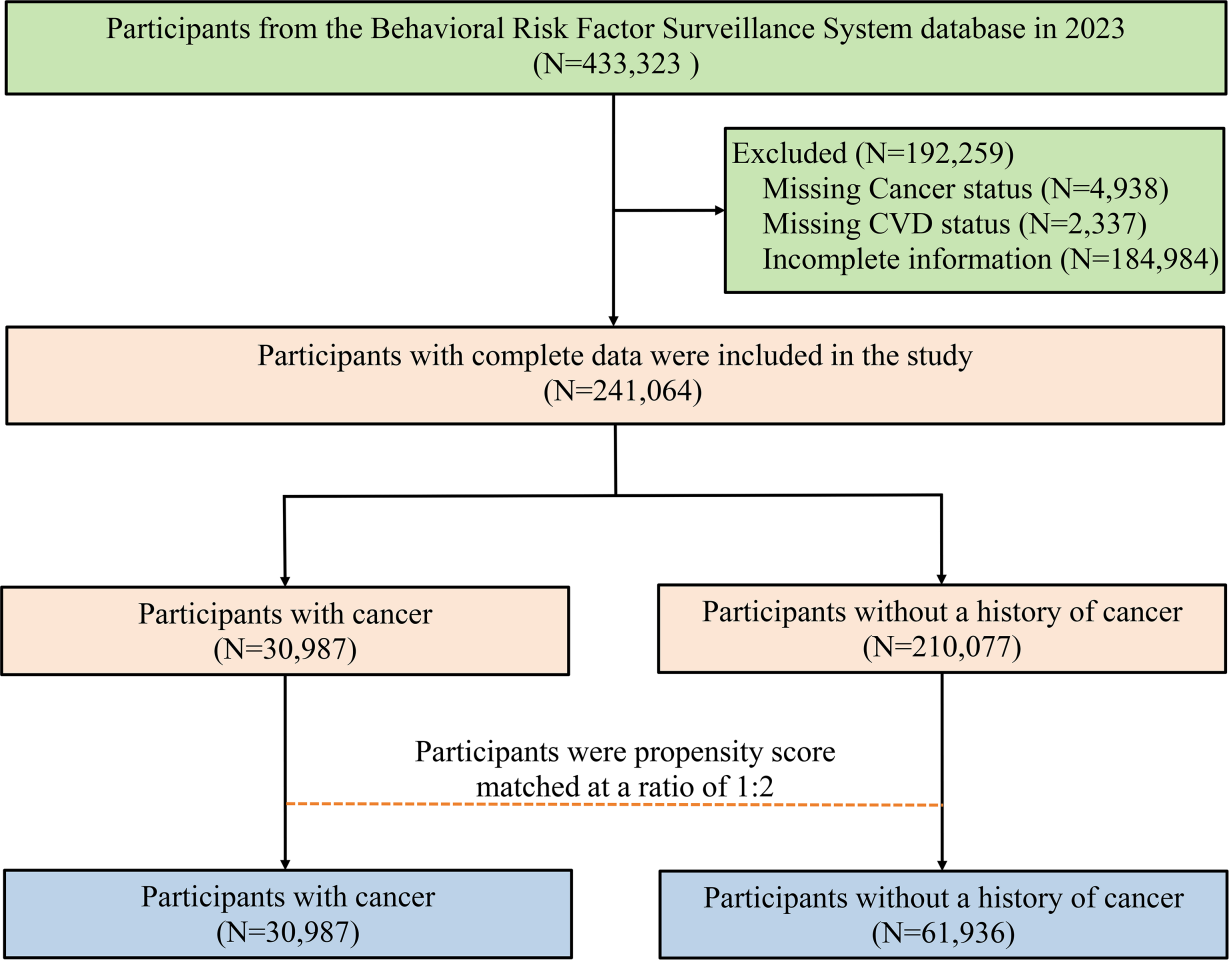
The flowchart of study participant screening.

Respondents aged ≥ 18 years with complete data from the 2023 BRFSS were included. Those with missing information on cancer status, CVD status, or key covariates were excluded. The detailed participant filtering process is shown in **Figure 1**.

### Study variables and outcomes

Key covariates included: (I) Demographic characteristics: age, sex, race, body mass index (BMI), marital status, residence; (II) Socioeconomic characteristics: educational attainment, income level; (III) Health status: history of stroke, dyslipidemia, hypertension, diabetes mellitus, chronic kidney disease (CKD), depression; (IV) Lifestyle factors: smoking status, Physical Activity Index (aerobic activity). The primary exposure variable was cancer status, defined by a positive response to the BRFSS question: “Have you ever been told by a doctor that you had melanoma or any other type of cancer?”. CVD status, defined as a composite endpoint comprising coronary heart disease, angina or coronary artery disease, myocardial infarction, or stroke, is the primary outcome measure.

### Statistical analysis

Participants were categorized into non-cancer and cancer groups based on their cancer status. PSM was performed to match cancer cases to non-cancer controls in a 1:2 ratio (method = “nearest”, distance = “logit”, caliper = 0.1). Matching effectiveness was systematically assessed using standardized mean differences (SMD) and other metrics (17). Univariate and multivariate logistic regression were employed to evaluate the correlation between cancer status (exposure) and CVD (outcome) before and after PSM. Multivariate logistic models adjusted for significant covariates. Subgroup analysis is utilized for populations with sufficient sample sizes. In subgroup analyses, binary logistic regression assessed the cancer-CVD association within specific populations, with age dichotomized by the median (< 71 vs. ≥ 71 years). Finally, within the cancer survivor population, univariate and multivariate logistic regression were employed to systematically analyze the risk factors of CVD in cancer survivors. Continuous variables are expressed as median with interquartile range (IQR) and compared using the Mann-Whitney U test. Categorical data were described by frequency and percentage, comparisons between groups were made with the chi-square test. R software (version 4.4.3) was used for all analyses and P < 0.05 was significant.

## Results

### Population

Data from the 2023 BRFSS survey covered 433,323 participants. Excluding participants with missing cancer status, CVD status, or key covariates, the final analytic sample consisted of 241,064 individuals. The selection flowchart is shown in **Figure 1**.

### Baseline characteristics

**Figure 2:**
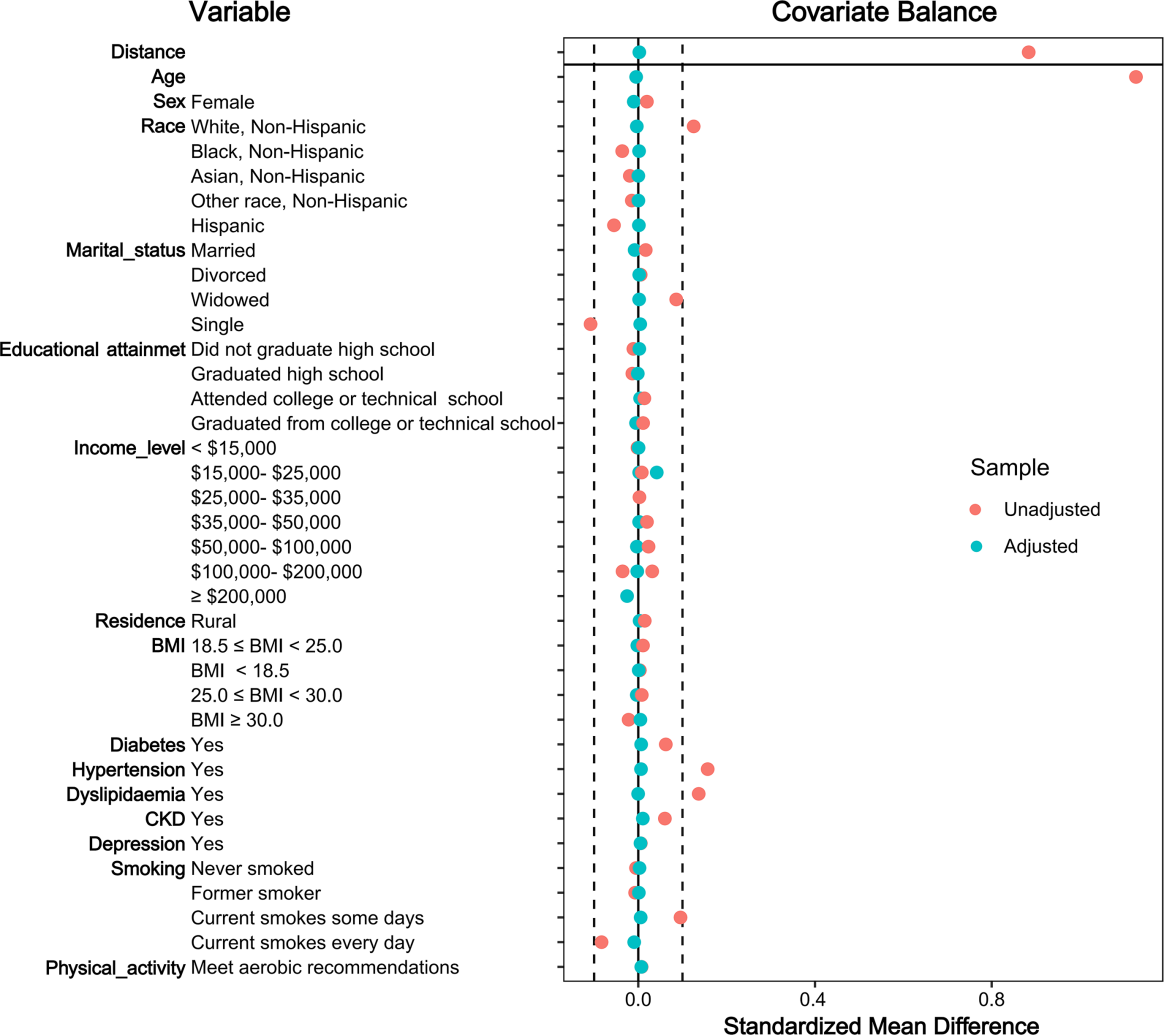
Standardized mean differences (SMDs) of variables before and after propensity score matching (PSM).

Based on cancer history, participants were categorized into two groups: Cancer group (n = 30,987, 12.9%) and non-cancer group (n = 210,077, 87.1%). Before PSM, significant differences were observed between groups regarding demographics, socioeconomic factors, health status, and lifestyle. After 1:2 PSM, 92,923 participants were included (Cancer group: n = 30,987; Non-cancer group: n = 61,936). All covariates demonstrated good balance post-matching (all SMD < 0.1; **Table 1**; **Figure 2**). Characteristics of the PSM-matched cohort were: median age 71 years (IQR 62-77, range 18-80); females 49,656 (53.4%); White 83,303 (89.6%); current smokers 84,289 (90.7%); not meeting aerobic physical activity guidelines 60,317 (64.9%).

**Table 1:** Demographic and clinical characteristics of the study population.

Demographic characteristics	Before PSM				After PSM			
	Cancer (N = 30987)	Non-Cancer (N = 210077)	SMD	P-value	Cancer (N = 30987)	Non-Cancer (N = 61936)	SMD	P-value
Age	71 (62,77)	58 (42,69)	1.1266	<0.001	71 (62,77)	71 (62,77)	-0.0048	0.641
Sex				<0.001				0.006
Male	14624 (47.19%)	103238 (49.14%)	-0.0195		14624 (47.19%)	28643 (46.25%)	0.0102	
Female	16363 (52.81%)	106839 (50.86%)	0.0195		16363 (52.81%)	33293 (53.75%)	-0.0102	
Race				<0.001				0.060
White, Non-Hispanic	27651 (89.23%)	161131 (76.70%)	0.1253		27651 (89.23%)	55652 (89.85%)	-0.0034	
Black, Non-Hispanic	1177 (3.80%)	15625 (7.44%)	-0.0364		1177 (3.80%)	2228 (3.60%)	0.0018	
Asian, Non-Hispanic	246 (0.79%)	5700 (2.71%)	-0.0192		246 (0.79%)	467 (0.75%)	0.0001	
Other race, Non-Hispanic	1089 (3.51%)	10471 (4.98%)	-0.0147		1089 (3.51%)	2076 (3.35%)	0.0002	
Hispanic	824 (2.66%)	17150 (8.16%)	-0.0550		824 (2.66%)	1513 (2.44%)	0.0013	
Marital status				<0.001				0.070
Married	17733 (57.23%)	116685 (55.54%)	0.0168		17733 (57.23%)	35987 (58.1%)	-0.0083	
Divorced	4898 (15.81%)	32078 (15.27%)	0.0054		4898 (15.81%)	9503 (15.34%)	0.0021	
Widowed	5600 (18.07%)	19929 (9.49%)	0.0859		5600 (18.07%)	11058 (17.85%)	0.0019	
Single	2756 (8.89%)	41385 (19.70%)	-0.1081		2756 (8.89%)	5388 (8.70%)	0.0043	
Educational attainment				<0.001				0.266
Did not graduate high school	968 (3.12%)	8902 (4.24%)	-0.0111		968 (3.12%)	1869 (3.02%)	0.0022	
Graduated high school	6414 (20.70%)	46302 (22.04%)	-0.0134		6414 (20.70%)	12738 (20.57%)	-0.0012	
Attended college or technical school	8584 (27.70%)	55312 (26.33%)	0.0137		8584 (27.70%)	16905 (27.29%)	0.0041	
Graduated from college or technical school	15021 (48.48%)	99561 (47.39%)	0.0108		15021 (48.48%)	30424 (49.12%)	-0.0051	
Income level				<0.001				0.157
< $15,000	1309 (4.22%)	9266 (4.41%)	-0.0019		1309 (4.22%)	2548 (4.11%)	0.0006	
$15,000- $25,000	2669 (8.61%)	16432 (7.82%)	0.0079		2669 (8.61%)	5176 (8.36%)	0.0020	
$25,000- $35,000	3503 (11.30%)	21181 (10.08%)	0.0122		3503 (11.30%)	6802 (10.98%)	0.0025	
$35,000- $50,000	4590 (14.81%)	26995 (12.85%)	0.0196		4590 (14.81%)	8981 (14.50%)	0.0019	
$50,000- $100,000	10445 (33.71%)	65954 (31.40%)	0.0231		10445 (33.71%)	21109 (34.08%)	-0.0033	
$100,000- $200,000	6457 (20.84%)	51258 (24.40%)	-0.0356		6457 (20.84%)	13200 (21.31%)	-0.0024	
≥ $200,000	2014 (6.50%)	18991 (9.04%)	-0.0254		2014 (6.50%)	4120 (6.65%)	-0.0013	
Residence				<0.001				0.084
Urban	26479 (85.45%)	182575 (86.91%)	-0.0146		26479 (85.45%)	53186 (85.87%)	-0.0029	
Rural	4508 (14.55%)	27502 (13.09%)	0.0146		4508 (14.55%)	8750 (14.13%)	0.0029	
BMI				<0.001				0.332
18.5 ≤ BMI < 25.0	8978 (28.97%)	58639 (27.91%)	0.0106		8978 (28.97%)	18162 (29.32%)	-0.0022	
BMI < 18.5	522 (1.68%)	2811 (1.34%)	0.0035		522 (1.68%)	1030 (1.66%)	0.0010	
25.0 ≤ BMI < 30.0	11482 (37.05%)	76190 (36.27%)	0.0079		11482 (37.05%)	23103 (37.30%)	-0.0035	
BMI ≥ 30.0	10005 (32.29%)	72437 (34.48%)	-0.0219		10005 (32.29%)	19641 (31.71%)	0.0047	
Diabetes				<0.001				<0.001
No	24823 (80.11%)	181371 (86.34%)	0.0623		24823 (80.11%)	50246 (81.13%)	-0.0065	
Yes	6164 (19.89%)	28706 (13.66%)	0.0623		6164 (19.89%)	11690 (18.87%)	0.0065	
Hypertension				<0.001				0.046
No	13410 (43.28%)	123899 (58.98%)	-0.1570		13410 (43.28%)	27231 (43.97%)	-0.0062	
Yes	17577 (56.72%)	86178 (41.02%)	0.1570		17577 (56.72%)	34705 (56.03%)	0.0062	
Dyslipidemia				<0.001				0.954
No	14283 (46.09%)	125556 (59.77%)	-0.1367		14283 (46.09%)	28536 (46.07%)	0.0003	
Yes	16704 (53.91%)	84521 (40.23%)	0.1367		16704 (53.91%)	33400 (53.93%)	-0.0003	
CKD				<0.001				<0.001
No	27809 (89.74%)	201173 (95.76%)	-0.0602		27809 (89.74%)	56167 (90.69%)	-0.0102	
Yes	3178 (10.26%)	8904 (4.24%)	0.0602		3178 (10.26%)	5769 (9.31%)	0.0102	
Depression				0.024				0.050
No	24297 (78.41%)	165903 (78.97%)	-0.0056		24297 (78.41%)	48910 (78.97%)	-0.0049	
Yes	6690 (21.59%)	44174 (21.03%)	0.0056		6690 (21.59%)	13026 (21.03%)	0.0049	
Smoking				<0.001				0.011
Never smoked	2226 (7.18%)	16167 (7.70%)	-0.0051		2226 (7.18%)	4223 (6.82%)	0.0027	
Former smoker	767 (2.48%)	6701 (3.19%)	-0.0071		767 (2.48%)	1418 (2.29%)	0.0012	
Current smokes some days	11731 (37.86%)	59467 (28.31%)	0.0955		11731 (37.86%)	23215 (37.48%)	0.0054	
Current smokes every day	16263 (52.48%)	127742 (60.81%)	-0.0832		16263 (52.48%)	33080 (53.41%)	-0.0093	
Physical activity				0.01				0.002
Did not meet aerobic recommendations	19898 (64.21%)	136463 (64.96%)	-0.0074		19898 (64.21%)	40419 (65.26%)	-0.0068	
Meet aerobic recommendations	11089 (35.79%)	73614 (35.04%)	0.0074		11089 (35.79%)	21517 (34.74%)	0.0068	

BMI, Body mass index; CKD, Chronic kidney disease; PSM, propensity score matching; SMD, Standardized Mean Difference.

### Univariate and multivariate logistic regression before and after PSM

**Figure 3:**
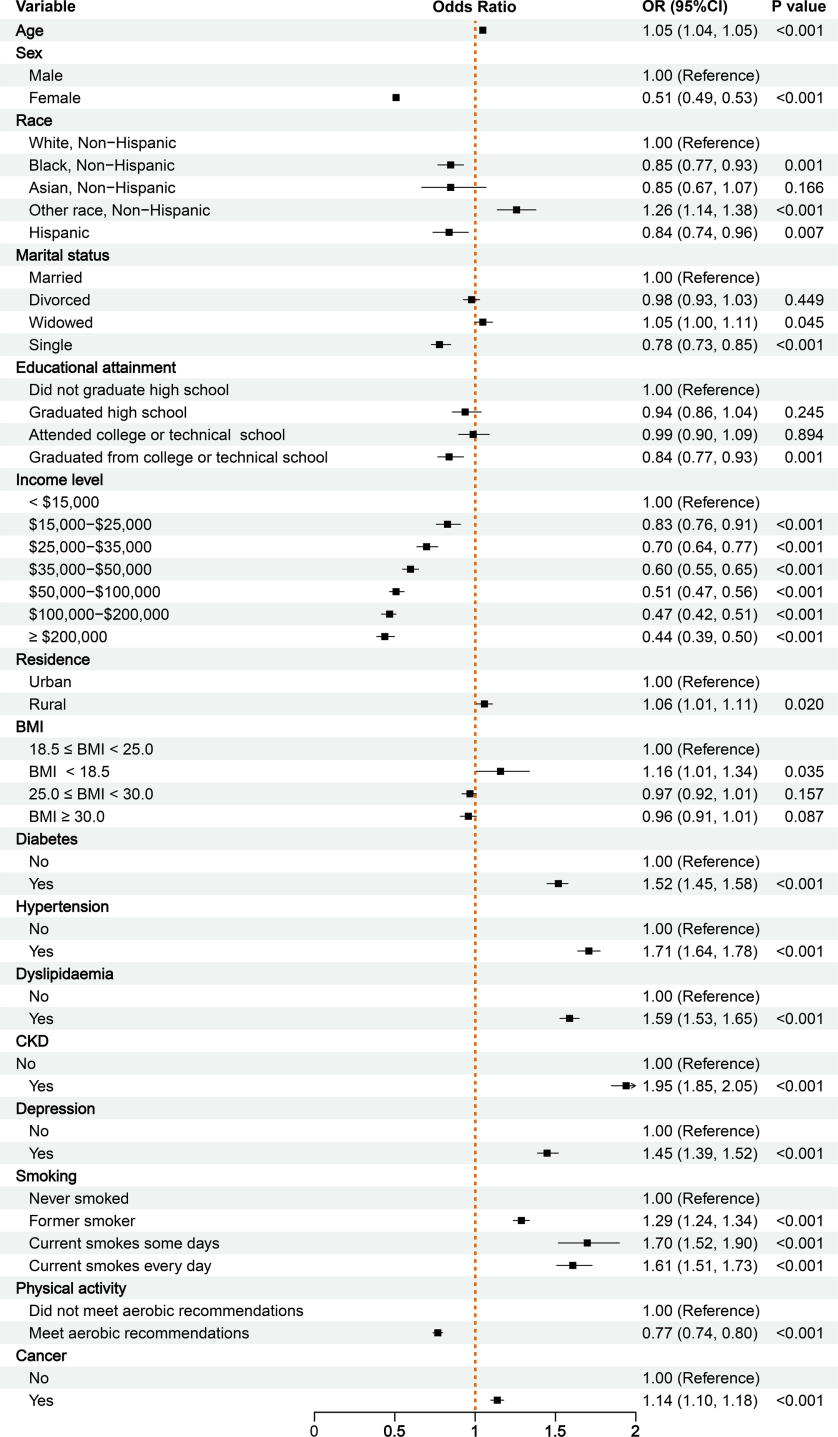
The forest plot of multivariate logistic regression analysis of cardiovascular disease risk between cancer survivors and non-cancer controls.

Within the total included population (N = 241,064), 28,785 participants (11.9%) had CVD. The prevalence of CVD was significantly higher in cancer patients than in non-cancer individuals (20.7% [6,423/30,987] vs. 10.7% [22,461/210,077], P < 0.001). Univariate logistic regression indicated a positive association between cancer and CVD risk (odds ratio [OR]: 2.14, 95% confidence interval [CI]: 2.08-2.21; P < 0.001). Adjusting for age, sex, and key covariates in multivariate logistic regression, cancer remains positively associated with CVD risk (OR = 1.16, 95% CI: 1.12-1.20, P < 0.001) (**Table 2**). To control for selection bias, 1:2 PSM was performed. Post-matching, 17,587 participants (18.9%) had CVD. The prevalence of CVD was significantly higher in cancer patients than in non-cancer individuals (20.7% [6,423/30,987] vs. 18.2% [11,265/61,936], P < 0.001). Both univariate and multivariate regression analyses within the matched cohort confirmed this association (Univariate: OR = 1.15, 95% CI: 1.11-1.19; Multivariate: OR = 1.14, 95% CI: 1.10-1.18; both P < 0.001) (**Figure 3**). This demonstrates that a history of cancer is positively associated with CVD risk, with a significant effect size persisting after multivariable adjustment and PSM.

**Table 2:** Univariate and multivariate logistic regression analyses of CVD risk factors before and after PSM.

Demographic characteristics	Before PSM				After PSM			
	Univariate logistic analysis		Multivariate logistic analysis		Univariate logistic analysis		Multivariate logistic analysis	
	OR (95% CI)	P-value	OR (95% CI)	P-value	OR (95% CI)	P-value	OR (95% CI)	P-value
Age	1.06 (1.06, 1.06)	<0.001	1.05 (1.05, 1.05)	<0.001	1.05 (1.05, 1.06)	<0.001	1.05 (1.04, 1.05)	<0.001
Sex								
Male	1.00 (Reference)		1.00 (Reference)		1.00 (Reference)		1.00 (Reference)	
Female	0.65 (0.64, 0.67)	<0.001	0.54 (0.53, 0.56)	<0.001	0.57 (0.55, 0.59)	<0.001	0.51 (0.49, 0.53)	<0.001
Race								
White, Non-Hispanic	1.00 (Reference)		1.00 (Reference)		1.00 (Reference)		1.00 (Reference)	
Black, Non-Hispanic	0.93 (0.89, 0.98)	0.005	0.94 (0.89, 1.00)	0.036	1.00 (0.92, 1.09)	0.968	0.85 (0.77, 0.93)	0.001
Asian, Non-Hispanic	0.42 (0.37, 0.46)	<0.001	0.81 (0.72, 0.92)	0.001	0.66 (0.53, 0.82)	<0.001	0.85 (0.67, 1.07)	0.166
Other race, Non-Hispanic	1.05 (1.00, 1.11)	0.062	1.23 (1.16, 1.31)	<0.001	1.29 (1.19, 1.41)	<0.001	1.26 (1.14, 1.38)	<0.001
Hispanic	0.50 (0.47, 0.53)	<0.001	0.82 (0.76, 0.87)	<0.001	0.78 (0.69, 0.87)	<0.001	0.84 (0.74, 0.96)	0.007
Marital status								
Married	1.00 (Reference)		1.00 (Reference)		1.00 (Reference)		1.00 (Reference)	
Divorced	1.46 (1.41, 1.51)	<0.001	0.99 (0.96, 1.03)	0.781	1.28 (1.23, 1.34)	<0.001	0.98 (0.93, 1.03)	0.449
Widowed	2.45 (2.37, 2.54)	<0.001	1.04 (1.00, 1.08)	0.073	1.67 (1.60, 1.74)	<0.001	1.05 (1.00, 1.11)	0.045
Single	0.57 (0.55, 0.60)	<0.001	0.86 (0.82, 0.9)	<0.001	0.81 (0.76, 0.87)	<0.001	0.78 (0.73, 0.85)	<0.001
Educational attainment								
Did not graduate high school	1.00 (Reference)		1.00 (Reference)		1.00 (Reference)		1.00 (Reference)	
Graduated high school	0.75 (0.71, 0.79)	<0.001	0.88 (0.83, 0.94)	<0.001	0.74 (0.68, 0.81)	<0.001	0.94 (0.86, 1.04)	0.245
Attended college or technical school	0.68 (0.64, 0.72)	<0.001	0.93 (0.87, 0.99)	0.022	0.66 (0.60, 0.72)	<0.001	0.99 (0.90, 1.09)	0.894
Graduated from college or technical school	0.42 (0.39, 0.44)	<0.001	0.78 (0.73, 0.83)	<0.001	0.44 (0.40, 0.48)	<0.001	0.84 (0.77, 0.93)	0.001
Income level								
< $15,000	1.00 (Reference)		1.00 (Reference)		1.00 (Reference)		1.00 (Reference)	
$15,000- $25,000	0.96 (0.91, 1.02)	0.224	0.82 (0.77, 0.87)	<0.001	0.97 (0.89, 1.06)	0.53	0.83 (0.76, 0.91)	<0.001
$25,000- $35,000	0.77 (0.73, 0.82)	<0.001	0.71 (0.66, 0.75)	<0.001	0.80 (0.74, 0.87)	<0.001	0.70 (0.64, 0.77)	<0.001
$35,000- $50,000	0.61 (0.58, 0.65)	<0.001	0.58 (0.55, 0.62)	<0.001	0.65 (0.60, 0.70)	<0.001	0.60 (0.55, 0.65)	<0.001
$50,000- $100,000	0.43 (0.41, 0.46)	<0.001	0.49 (0.46, 0.52)	<0.001	0.49 (0.45, 0.52)	<0.001	0.51 (0.47, 0.56)	<0.001
$100,000- $200,000	0.28 (0.26, 0.29)	<0.001	0.44 (0.41, 0.47)	<0.001	0.34 (0.32, 0.37)	<0.001	0.47 (0.42, 0.51)	<0.001
≥ $200,000	0.19 (0.18, 0.21)	<0.001	0.39 (0.36, 0.43)	<0.001	0.27 (0.24, 0.30)	<0.001	0.44 (0.39, 0.50)	<0.001
Residence								
Urban	1.00 (Reference)		1.00 (Reference)		1.00 (Reference)		1.00 (Reference)	
Rural	1.35 (1.31, 1.40)	<0.001	1.04 (1.00, 1.08)	0.036	1.21 (1.16, 1.27)	<0.001	1.06 (1.01, 1.11)	0.020
BMI								
18.5 ≤ BMI < 25.0	1.00 (Reference)		1.00 (Reference)		1.00 (Reference)		1.00 (Reference)	
BMI < 18.5	1.38 (1.25, 1.53)	<0.001	1.23 (1.10, 1.38)	<0.001	1.23 (1.08, 1.40)	0.002	1.16 (1.01, 1.34)	0.035
25.0 ≤ BMI < 30.0	1.23 (1.19, 1.27)	<0.001	0.98 (0.94, 1.01)	0.177	1.20 (1.15, 1.25)	<0.001	0.97 (0.92, 1.01)	0.157
BMI ≥ 30.0	1.40 (1.35, 1.44)	<0.001	0.99 (0.96, 1.03)	0.649	1.33 (1.27, 1.39)	<0.001	0.96 (0.91, 1.01)	0.087
Diabetes								
No	1.00 (Reference)		1.00 (Reference)		1.00 (Reference)		1.00 (Reference)	
Yes	3.39 (3.29, 3.48)	<0.001	1.59 (1.54, 1.64)	<0.001	2.46 (2.37, 2.56)	<0.001	1.52 (1.45, 1.58)	<0.001
Hypertension								
No	1.00 (Reference)		1.00 (Reference)		1.00 (Reference)		1.00 (Reference)	
Yes	4.16 (4.04, 4.27)	<0.001	1.86 (1.81, 1.92)	<0.001	2.82 (2.71, 2.92)	<0.001	1.71 (1.64, 1.78)	<0.001
Dyslipidemia								
No	1.00 (Reference)		1.00 (Reference)		1.00 (Reference)		1.00 (Reference)	
Yes	2.97 (2.90, 3.05)	<0.001	1.65 (1.60, 1.70)	<0.001	2.13 (2.05, 2.2)	<0.001	1.59 (1.53, 1.65)	<0.001
CKD								
No	1.00 (Reference)		1.00 (Reference)		1.00 (Reference)		1.00 (Reference)	
Yes	4.48 (4.31, 4.66)	<0.001	2.04 (1.95, 2.13)	<0.001	3.01 (2.88, 3.16)	<0.001	1.95 (1.85, 2.05)	<0.001
Depression								
No	1.00 (Reference)		1.00 (Reference)		1.00 (Reference)		1.00 (Reference)	
Yes	1.38 (1.34, 1.42)	<0.001	1.51 (1.47, 1.57)	<0.001	1.36 (1.31, 1.41)	<0.001	1.45 (1.39, 1.52)	<0.001
Smoking								
Never smoked	1.00 (Reference)		1.00 (Reference)		1.00 (Reference)		1.00 (Reference)	
Former smoker	2.09 (2.04, 2.15)	<0.001	1.34 (1.30, 1.38)	<0.001	1.68 (1.63, 1.74)	<0.001	1.29 (1.24, 1.34)	<0.001
Current smokes some days	1.83 (1.71, 1.96)	<0.001	1.72 (1.60, 1.85)	<0.001	1.75 (1.58, 1.93)	<0.001	1.70 (1.52, 1.90)	<0.001
Current smokes every day	2.17 (2.08, 2.26)	<0.001	1.69 (1.61, 1.77)	<0.001	1.83 (1.72, 1.95)	<0.001	1.61 (1.51, 1.73)	<0.001
Physical activity								
Did not meet aerobic recommendations	1.00 (Reference)		1.00 (Reference)		1.00 (Reference)		1.00 (Reference)	
Meet aerobic recommendations	0.62 (0.60, 0.63)	<0.001	0.80 (0.78, 0.82)	<0.001	0.60 (0.58, 0.62)	<0.001	0.77 (0.74, 0.80)	<0.001
Cancer								
No	1.00 (Reference)		1.00 (Reference)		1.00 (Reference)		1.00 (Reference)	
Yes	2.14 (2.08, 2.21)	<0.001	1.16 (1.12, 1.20)	<0.001	1.15 (1.11, 1.19)	<0.001	1.14 (1.10, 1.18)	<0.001

BMI, Body mass index; CI, Confidence interval; CKD, Chronic kidney disease; CVD, Cardiovascular disease; OR, Odds ratio; PSM, propensity score matching.

### Subgroup analysis

**Figure 4:**
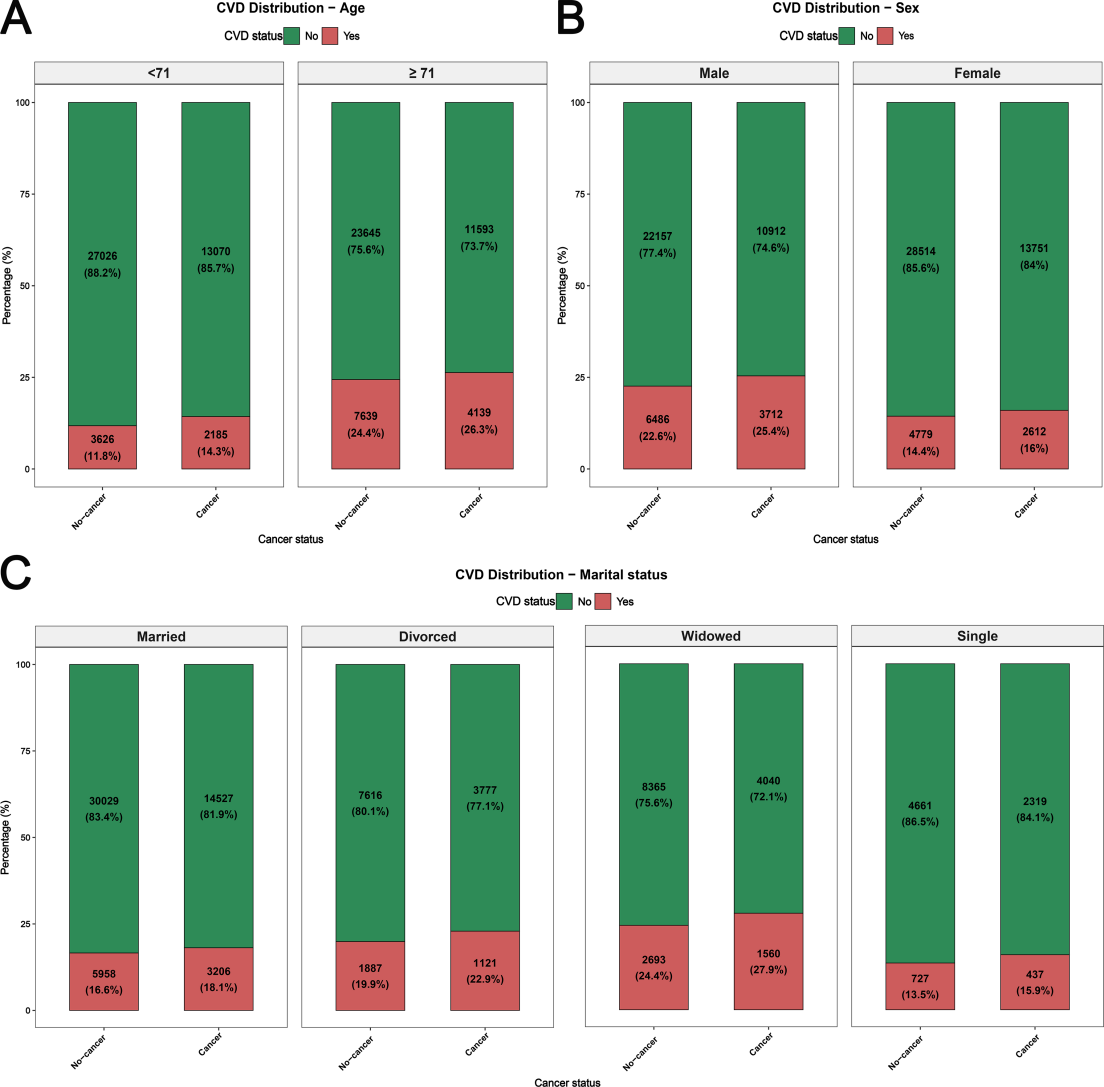
Cardiovascular disease in cancer survivors vs non-cancer individuals: Case numbers and proportions across clinical subgroups. **(A)** Age; **(B)** Sex; **(C)** Marital status.

**Figure 5:**
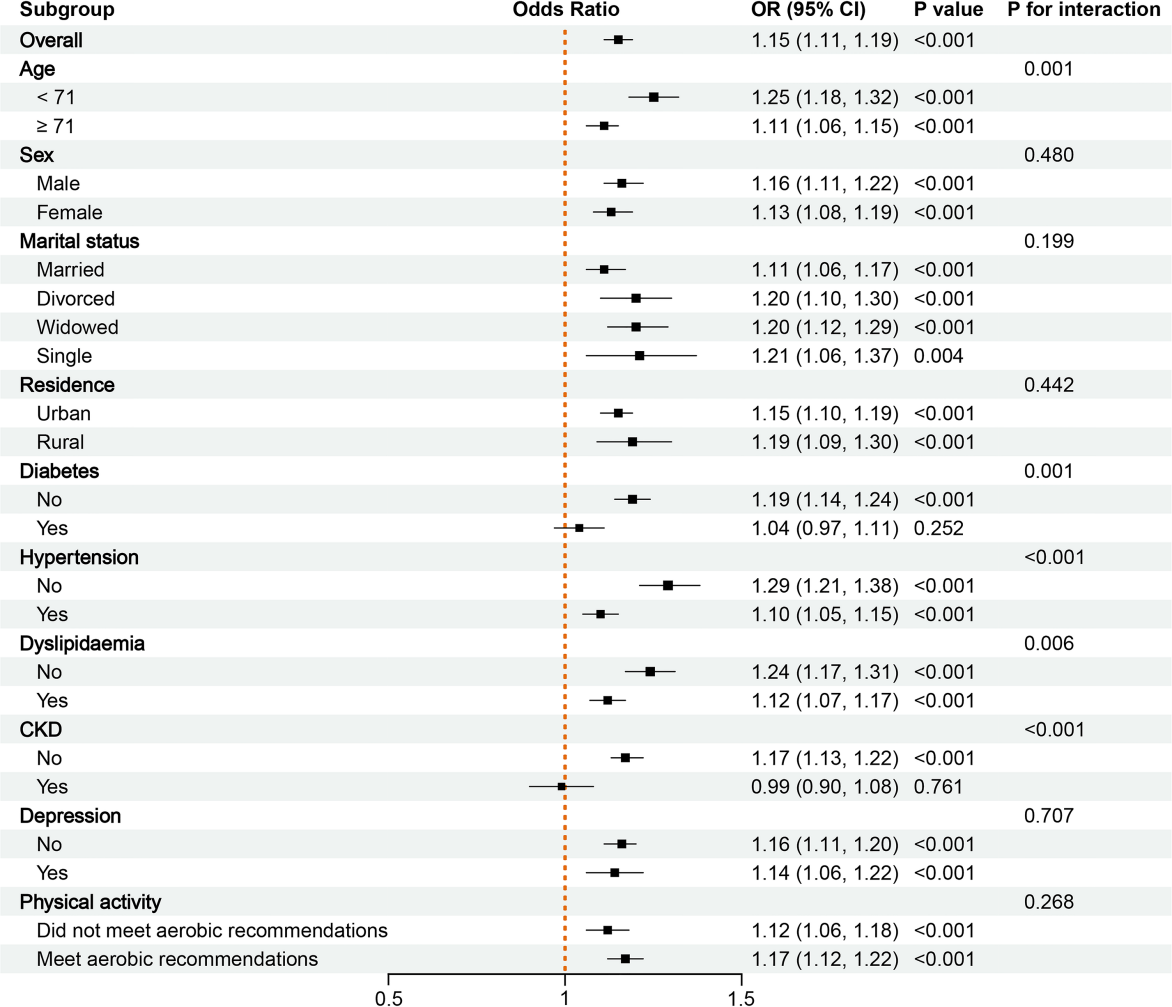
The forest plot of subgroup analysis of cardiovascular disease risk between cancer survivors and non-cancer individuals after propensity score matching (PSM).

We conducted a subgroup analysis on populations with sufficient sample sizes. Subgroup analyses assessed the consistency of the association between cancer survivorship and CVD. Bar charts illustrated CVD case numbers and proportions in cancer survivors vs. non-cancer individuals across subgroups (**Figure 4**; **Supplementary Figures S1-S2**). Forest plot results indicated that among 22 subgroups, the ORs were significantly greater than 1 (P < 0.05) in all except for those with diabetes and CKD, where no significant statistical difference was found. Notably, no subgroup exhibited a statistically significant inverse association (OR < 1, P < 0.05) (**Supplementary Table S1**; **Figure 5**).

### Risk factors for CVD in cancer survivors

**Figure 6:**
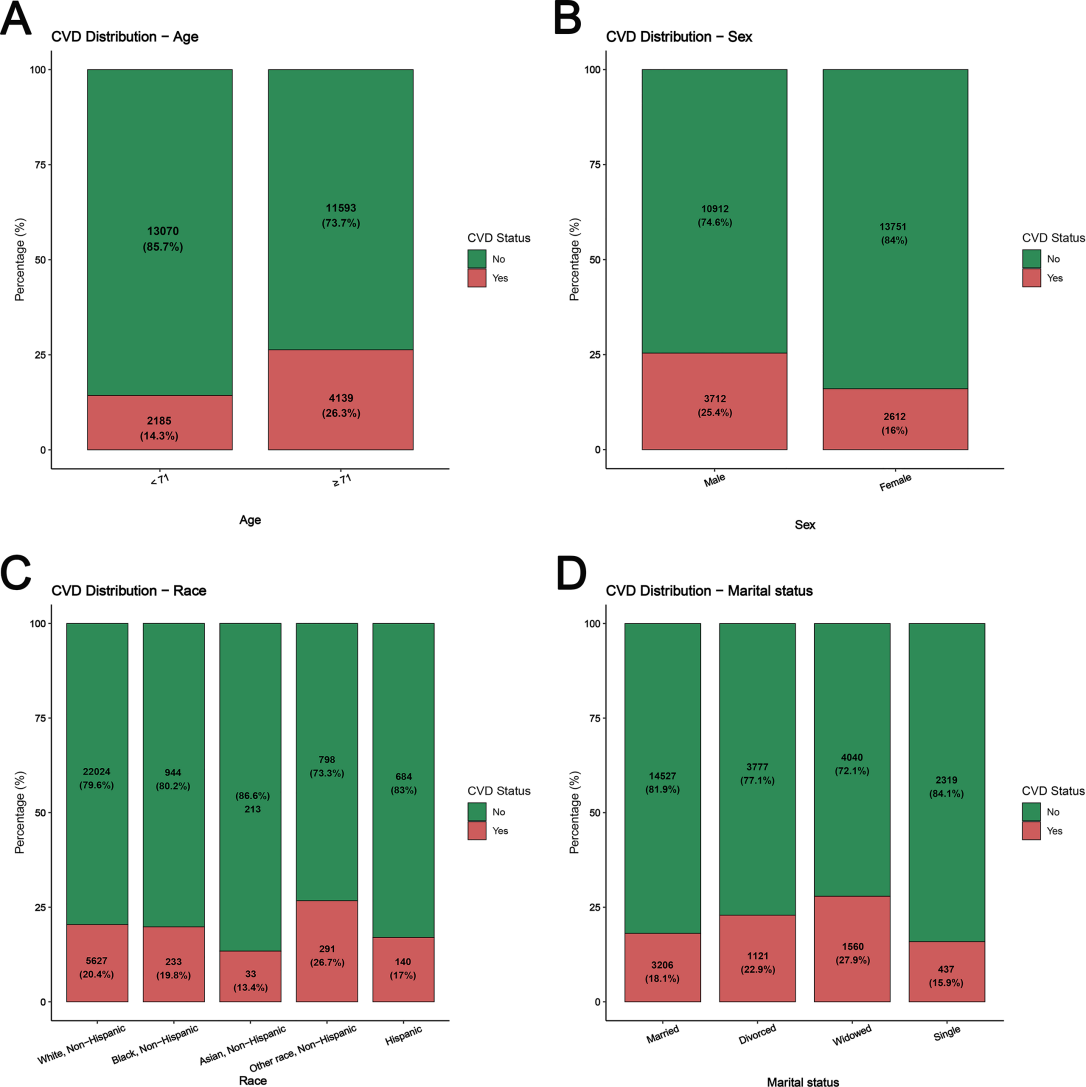
Cardiovascular disease in cancer survivors: Case numbers and proportions across clinical subgroups. **(A)** Age; **(B)** Sex; **(C)** Race; **(D)** Marital status.

The bar charts displayed CVD case numbers and proportions across different characteristics within the cancer survivor group (**Figure 6**; **Supplementary Figures S3-S5**). Univariate and multivariate logistic regression analyses were performed specifically within cancer survivors. Univariate logistic regression results indicated that all 15 included covariates were significantly associated with CVD occurrence (P < 0.05) (**Table 3**). The multivariate model further showed that among cancer survivors, increased CVD risk was significantly associated with older age (OR = 1.04, 95% CI: 1.03-1.04, P < 0.001), race categorized as “Other” (OR = 1.41, 95% CI: 1.21-1.64, P < 0.001), and the presence of comorbidities: hyperglycemia/diabetes (OR = 1.46, 95% CI: 1.36-1.56, P < 0.001), hypertension (OR = 1.63, 95% CI: 1.53-1.75, P < 0.001), dyslipidemia (OR = 1.52, 95% CI: 1.43-1.62, P < 0.001), CKD (OR = 1.85, 95% CI: 1.70-2.01, P < 0.001), depression (OR = 1.46, 95% CI: 1.36-1.57, P < 0.001), and smoking. Reduced CVD risk was associated with female sex (OR = 0.52, 95% CI: 0.49-0.56, P < 0.001), being single (OR = 0.82, 95% CI: 0.72-0.92, P < 0.001), higher income level, urban residence, elevated BMI, and meeting aerobic physical activity guidelines (OR = 0.78, 95% CI: 0.73-0.83, P < 0.001). Educational level was not significantly associated with CVD (P > 0.05) (**Supplementary Figure S6**).

**Table 3:** Univariate and multivariate logistic regression analyses of CVD risk factors in cancer survivors.

Demographic characteristics	Univariate logistic analysis		Multivariate logistic analysis	
	OR (95% CI)	P-value	OR (95% CI)	P-value
Age	1.05 (1.04, 1.05)	<0.001	1.04 (1.03, 1.04)	<0.001
Sex				
Male	1.00 (Reference)		1.00 (Reference)	
Female	0.56 (0.53, 0.59)	<0.001	0.52 (0.49, 0.56)	<0.001
Race				
White, Non-Hispanic	1.00 (Reference)		1.00 (Reference)	
Black, Non-Hispanic	0.97 (0.83, 1.12)	0.644	0.83 (0.71, 0.97)	0.018
Asian, Non-Hispanic	0.61 (0.42, 0.88)	0.008	0.86 (0.58, 1.26)	0.438
Other race, Non-Hispanic	1.43 (1.24, 1.64)	<0.001	1.41 (1.21, 1.64)	<0.001
Hispanic	0.80 (0.67, 0.96)	0.018	0.91 (0.74, 1.11)	0.349
Marital status				
Married	1.00 (Reference)		1.00 (Reference)	
Divorced	1.34 (1.25, 1.45)	<0.001	1.04 (0.95, 1.14)	0.388
Widowed	1.75 (1.63, 1.88)	<0.001	1.15 (1.06, 1.25)	0.001
Single	0.85 (0.77, 0.95)	0.005	0.82 (0.72, 0.92)	0.001
Educational attainment				
Did not graduate high school	1.00 (Reference)		1.00 (Reference)	
Graduated high school	0.75 (0.64, 0.87)	<0.001	0.96 (0.82, 1.13)	0.661
Attended college or technical school	0.64 (0.55, 0.74)	<0.001	0.97 (0.83, 1.14)	0.749
Graduated from college or technical school	0.45 (0.39, 0.52)	<0.001	0.87 (0.74, 1.03)	0.108
Income level				
< $15,000	1.00 (Reference)		1.00 (Reference)	
$15,000- $25,000	0.94 (0.82, 1.08)	0.396	0.78 (0.67, 0.91)	0.001
$25,000- $35,000	0.77 (0.67, 0.88)	<0.001	0.67 (0.58, 0.78)	<0.001
$35,000- $50,000	0.64 (0.56, 0.73)	<0.001	0.58 (0.50, 0.67)	<0.001
$50,000- $100,000	0.46 (0.41, 0.53)	<0.001	0.47 (0.41, 0.55)	<0.001
$100,000- $200,000	0.35 (0.30, 0.40)	<0.001	0.43 (0.37, 0.51)	<0.001
≥ $200,000	0.26 (0.21, 0.31)	<0.001	0.39 (0.32, 0.48)	<0.001
Residence				
Urban	1.00 (Reference)		1.00 (Reference)	
Rural	1.24 (1.15, 1.33)	<0.001	1.09 (1.00, 1.18)	0.038
BMI				
18.5 ≤ BMI < 25.0	1.00 (Reference)		1.00 (Reference)	
BMI < 18.5	1.19 (0.96, 1.47)	0.120	1.10 (0.87, 1.38)	0.440
25.0 ≤ BMI < 30.0	1.13 (1.05, 1.21)	0.001	0.92 (0.85, 0.99)	0.025
BMI ≥ 30.0	1.20 (1.12, 1.29)	<0.001	0.88 (0.81, 0.95)	0.001
Diabetes				
No	1.00 (Reference)		1.00 (Reference)	
Yes	2.25 (2.12, 2.4)	<0.001	1.46 (1.36, 1.56)	<0.001
Hypertension				
No	1.00 (Reference)		1.00 (Reference)	
Yes	2.54 (2.39, 2.70)	<0.001	1.63 (1.53, 1.75)	<0.001
Dyslipidemia				
No	1.00 (Reference)		1.00 (Reference)	
Yes	1.99 (1.88, 2.11)	<0.001	1.52 (1.43, 1.62)	<0.001
CKD				
No	1.00 (Reference)		1.00 (Reference)	
Yes	2.69 (2.49, 2.91)	<0.001	1.85 (1.70, 2.01)	<0.001
Depression				
No	1.00 (Reference)		1.00 (Reference)	
Yes	1.34 (1.26, 1.43)	<0.001	1.46 (1.36, 1.57)	<0.001
Smoking				
Never smoked	1.00 (Reference)		1.00 (Reference)	
Former smoker	1.65 (1.56, 1.75)	<0.001	1.28 (1.20, 1.37)	<0.001
Current smokes some days	1.82 (1.54, 2.15)	<0.001	1.69 (1.41, 2.03)	<0.001
Current smokes every day	2.03 (1.83, 2.24)	<0.001	1.69 (1.51, 1.90)	<0.001
Physical activity				
Did not meet aerobic recommendations	1.00 (Reference)		1.00 (Reference)	
Meet aerobic recommendations	0.62 (0.58, 0.65)	<0.001	0.78 (0.73, 0.83)	<0.001

BMI, Body mass index; CI, Confidence interval; CKD, Chronic kidney disease; CVD, Cardiovascular disease; OR, Odds ratio.

## Discussion

Cancer and CVD are the major causes of death globally (18, 19). In recent years, improvements in diagnostic and therapeutic technologies have markedly extended survival, yielding a growing cohort of cancer survivors (20). Substantial epidemiological evidence indicates that CVD is the leading cause of death among cancer survivors, excluding cancer as a cause of death (21), international cardiology and oncology societies increasingly recognize the clinical significance of CVD in cancer survivors (22). However, the relationship between malignant tumors and the risk of CVD remains debated (11).

This study, based on the U.S. BRFSS database, employed PSM to assess whether cancer survivors face a high risk of CVD. Additionally, we analyzed risk factors for CVD development in cancer survivors. After rigorous adjustment for confounders and PSM, our study found that cancer survivors face a 10% to 18% increased risk of developing CVD, consistent with most prior studies reporting elevated CVD risk in this population, particularly among those treated with chemotherapy or radiotherapy (23, 24). In contrast, studies reporting an inverse association between cancer and CVD primarily involved specific cancer types or unique subpopulations (25, 26). Although we did not analyze CVD risk in survivors of specific cancer types—a limitation that future research should address—we examined CVD risk in cancer survivors across different demographic subgroups.

Subgroup analyses further validated the consistency of findings across different populations. Among 22 subgroups, 20 demonstrated a positive association between cancer status and CVD risk, reinforcing the robustness of this association. Notably, no significant association was found in diabetes and CKD subgroups, where the extremely high baseline CVD risk may overshadow any additional impact from cancer (27, 28). Critically, no subgroups showed an inverse association between cancer and CVD risk, a finding corroborated by recent research (29).

The mechanisms linking cancer to elevated CVD risk are multifaceted. Based on our results and prior studies, we propose several explanations. Shared risk factors and pathogenic pathways, such as aging, hyperglycemia, alcohol use, smoking, and physical inactivity, may contribute to the increased risk (30). Additionally, cardiac toxicity directly induced by cancer treatments may also play a role, such as those associated with chemotherapy, radiotherapy, and immunotherapy (31). Cancer-associated hypercoagulability, increasing the risk of arterial and venous thrombosis (32), and psychological sequelae like stress, depression, and anxiety post-cancer diagnosis and treatment may also independently elevate CVD risk (33, 34). Of course, as this study is cross-sectional, the association between cancer history and CVD risk may reflect residual confounding, mediation by unmeasured treatment-related factors, or survival bias. The observed higher CVD prevalence among cancer patients may be attributable to age and traditional risk factors; cancer history largely signifies accumulated cardiovascular risk. Therefore, future longitudinal studies are needed to assess temporality and causality.

To identify high-risk subgroups and inform CVD prevention strategies, we analyzed risk factors among cancer survivors. Older age and male sex were related to an elevated risk of CVD, aligning with classical CVD epidemiology (35). Higher income correlated with lower CVD risk, likely due to better access to cancer treatment, CVD screening, and therapies (36, 37). Conversely, low income exacerbates financial toxicity, especially in rural areas where access to healthcare is limited (38). Cardiometabolic comorbidities (including hypertension, dyslipidemia, diabetes, and chronic kidney disease) represent shared risk factors between cancer and CVD. For instance, hypertension induces oxidative stress on arterial walls, a primary mechanism influencing atherosclerosis development (39), while also exhibiting a particularly strong association with renal cell carcinoma (40). Atherosclerosis originates from excessive accumulation of low-density lipoprotein beneath the endothelium (41), while high-fat intake may promote bile acid-mediated carcinogenesis and increase colorectal cancer risk (42). Among cancer survivors, these comorbidities further elevate the risk of cardiovascular disease (43, 44). Depression and other mental health factors are also associated with a higher incidence of CVD (45). Lifestyle factors played a significant role in CVD risk among cancer survivors. Current and former smokers faced elevated CVD risk, as smoking is a shared etiology for cancer and CVD (46). Adequate aerobic exercise reduced CVD risk by 22%, potentially mitigating chemotherapy-induced cardiotoxicity and coronary artery disease. Surprisingly, overweight/obese cancer survivors show an inverse relationship with CVD risk—a finding inconsistent with conventional views that warrants further investigation in prospective studies (47, 48).

The significantly elevated CVD risk among cancer survivors carries important clinical implications. Multidisciplinary collaboration between oncologists and cardiologists is essential for systematic risk management. Regular CVD risk stratification and monitoring can help identify high-risk individuals for early intervention. Lifestyle modifications, such as smoking cessation and structured aerobic exercise, may reduce CVD incidence and improve long-term quality of life.

This study benefits from several key strengths: 1) A large sample size, enhancing result accuracy; 2) Utilization of multiple analytical approaches (multivariable adjustment, PSM) to robustly demonstrate higher CVD risk among cancer survivors compared with non-cancer controls; 3) Identification of specific CVD risk and protective factors within the cancer survivor population, informing targeted prevention and management. Limitations include: 1) Based on telephone survey data, this retrospective cross-sectional study lacks temporal sequencing, making it difficult to establish causality, and may rely on participants’ recall and be subject to participant bias; 2) Absence of detailed information on cancer stage, type, and specific treatment regimens limits the ability to assess how these factors influence long-term cardiovascular outcomes. 3) Lack of longitudinal follow-up data within BRFSS prevents assessment of incident CVD risk over time; 4) The predominantly US-based population may limit applicability to other countries and ethnicities. Collectively, these limitations restrict the clinical applicability of the findings in modern cardio-oncology practice. Future prospective studies should include more detailed risk variables and incident CVD events during follow-up period to corroborate the robustness of our results.

## Conclusion

There is a significant positive correlation between cancer and CVD risk, and this finding is confirmed across the majority of clinical subgroups. In cancer survivors, the main positive associations with CVD risk are age, hypertension, diabetes, dyslipidemia, CKD, and smoking. This study provides evidence for strengthening CVD risk assessment and prevention efforts among cancer survivors. Due to the study’s limitations, large prospective studies are warranted for further validation.

## Data Availability

The original contributions presented in the study are included in the article/**Supplementary Material**. Further inquiries can be directed to the corresponding author.

## Supplementary material

**Supplementary Figure S1** Cardiovascular disease in cancer survivors vs non-cancer individuals: Case numbers and proportions across clinical subgroups. **(A)** Residence; **(B)** Diabetes; **(C)** Hypertension; **(D)** Dyslipidemia.

**Supplementary Figure S2** Cardiovascular disease in cancer survivors vs non-cancer individuals: Case numbers and proportions across clinical subgroups. **(A)** Chronic kidney disease (CKD); **(B)** Depression; **(C)** Physical activity.

**Supplementary Figure S3** Cardiovascular disease in cancer survivors: Case numbers and proportions across clinical subgroups. **(A)** Educational attainment; **(B)** Income level; **(C)** Residence; **(D)** Body mass index (BMI).

**Supplementary Figure S4** Cardiovascular disease in cancer survivors: Case numbers and proportions across clinical subgroups. **(A)** Diabetes; **(B)** Hypertension; **(C)** Dyslipidemia; **(D)** Chronic kidney disease (CKD).

**Supplementary Figure S5** Cardiovascular disease in cancer survivors: Case numbers and proportions across clinical subgroups. **(A)** Depression; **(B)** Smoking; **(C)** Physical activity.

**Supplementary Figure S6** The forest plot of multivariate logistic regression analysis of cardiovascular disease risk in cancer survivors.

**Supplementary Table S1** Subgroup analysis of cardiovascular disease risk in cancer survivors versus non-cancer individuals after propensity score matching (PSM).
