# Supplementary figures and images for: Association between cancer and cardiovascular disease risk: a cross-sectional study of 241,064 individuals
Source: Front Oncol. 2026 Feb 4;16:1734601. doi: 10.3389/fonc.2026.1734601 (PMC12913180; doi:10.3389/fonc.2026.1734601)

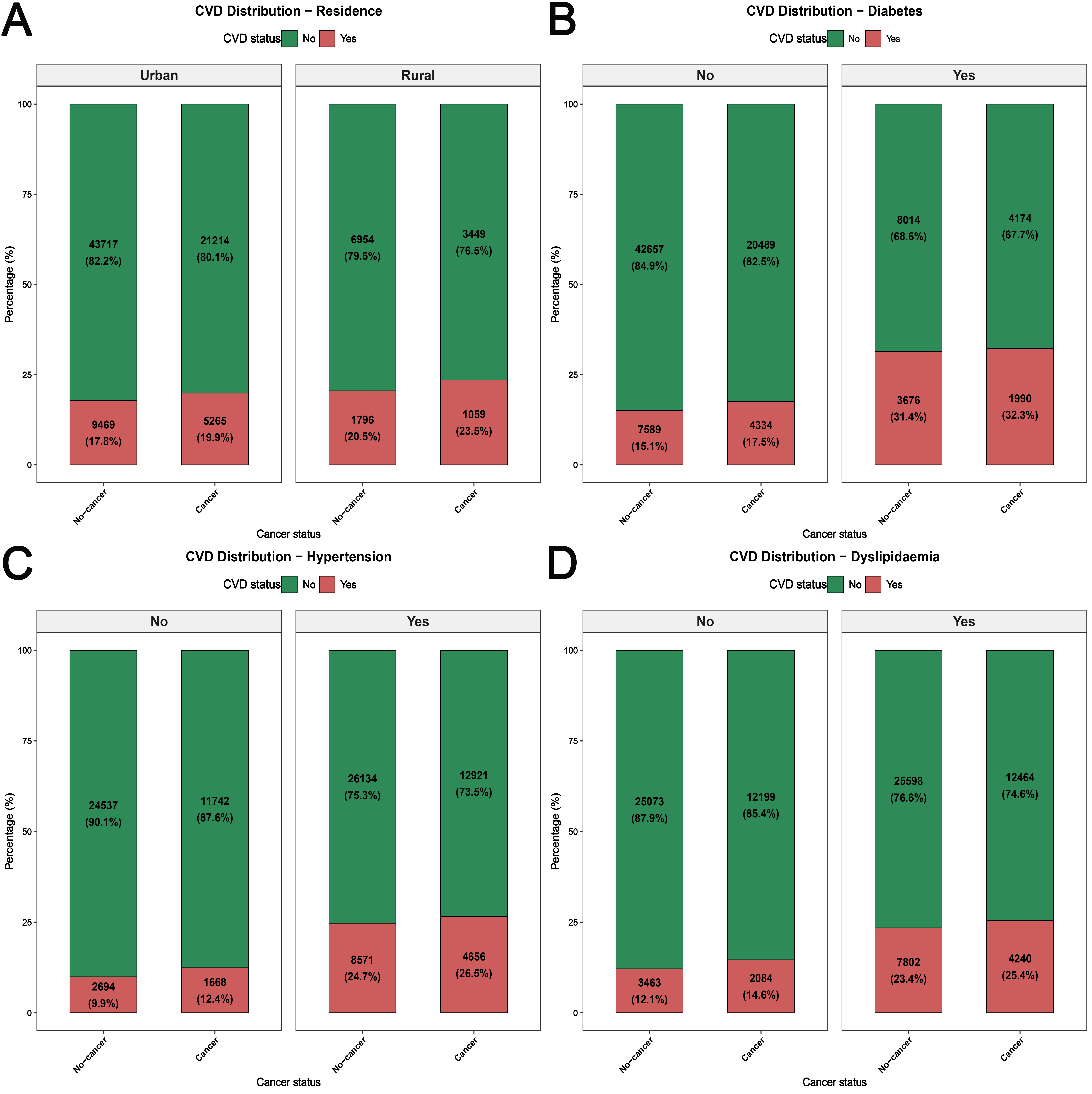

Supplement: Supplementary file 1 — Supplementary Figure S1 Cardiovascular disease in cancer survivors vs non-cancer individuals: Case numbers and proportions across clinical subgroups. (A) Residence; (B) Diabetes; (C) Hypertension; (D) Dyslipidemia. [file Image1.tif]

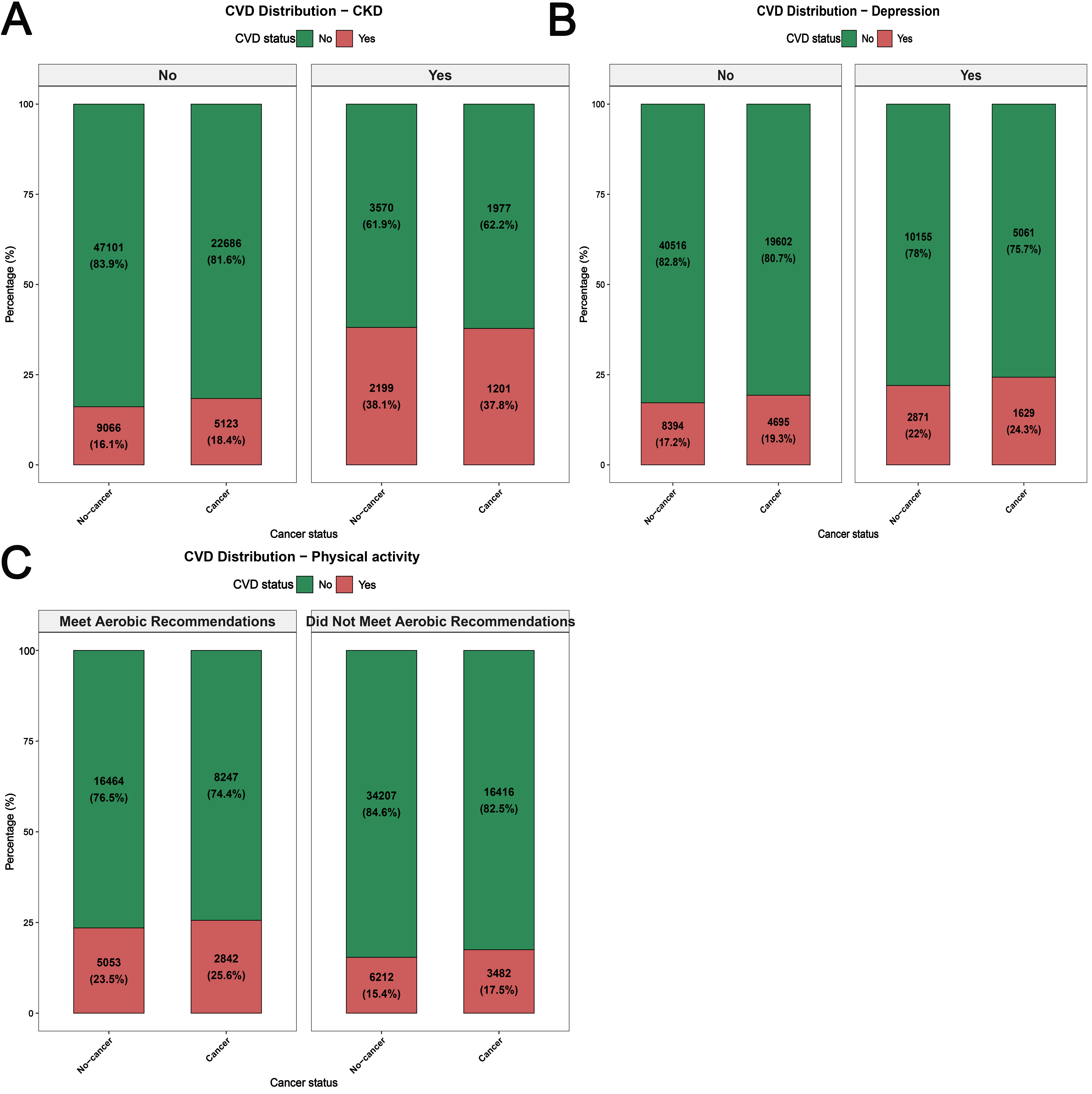

Supplement: Supplementary file 2 — Supplementary Figure S2 Cardiovascular disease in cancer survivors vs non-cancer individuals: Case numbers and proportions across clinical subgroups. (A) Chronic kidney disease (CKD); (B) Depression; (C) Physical activity. [file Image2.tif]

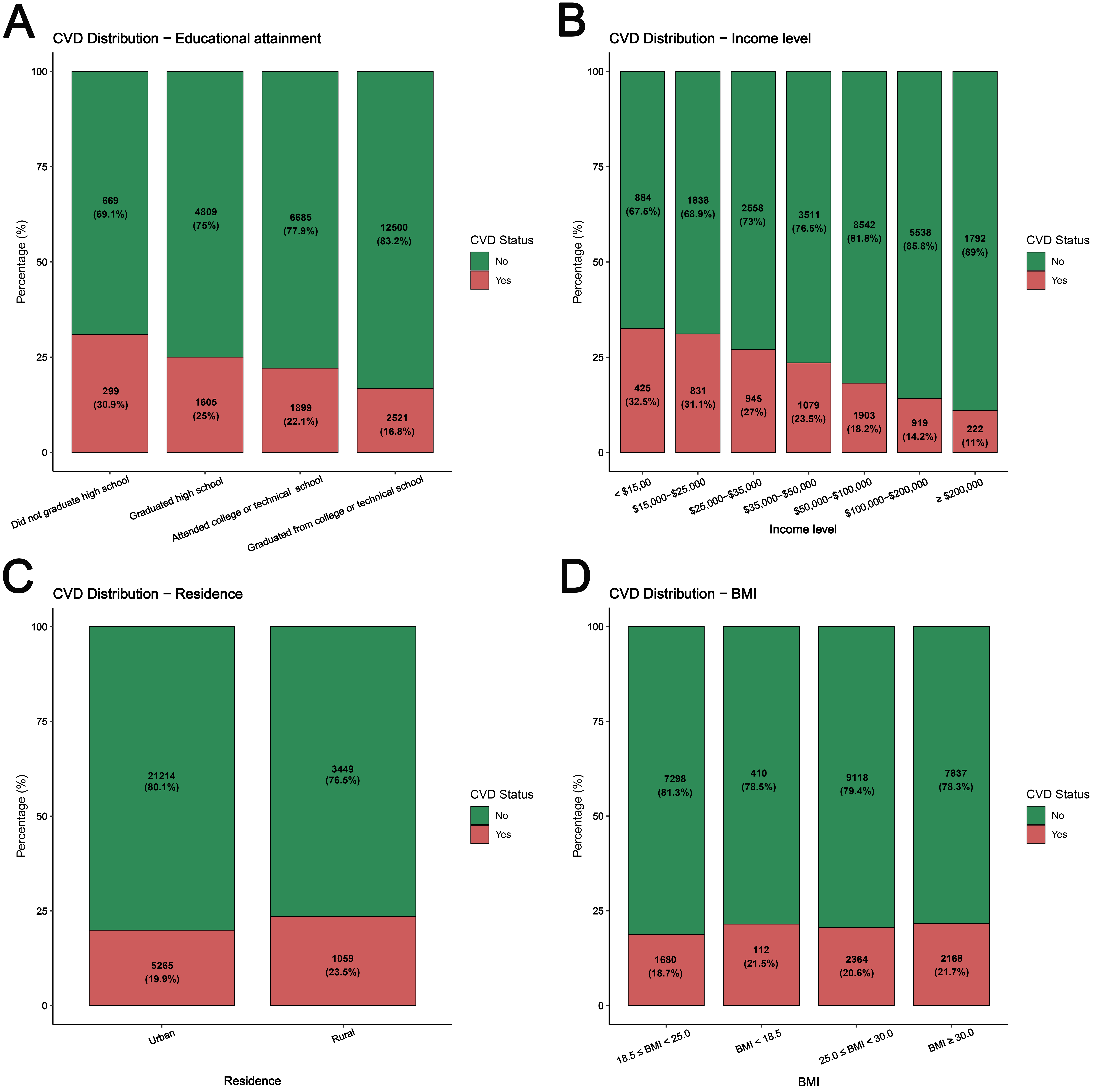

Supplement: Supplementary file 3 — Supplementary Figure S3 Cardiovascular disease in cancer survivors: Case numbers and proportions across clinical subgroups. (A) Educational attainment; (B) Income level; (C) Residence; (D) Body mass index (BMI). [file Image3.tif]

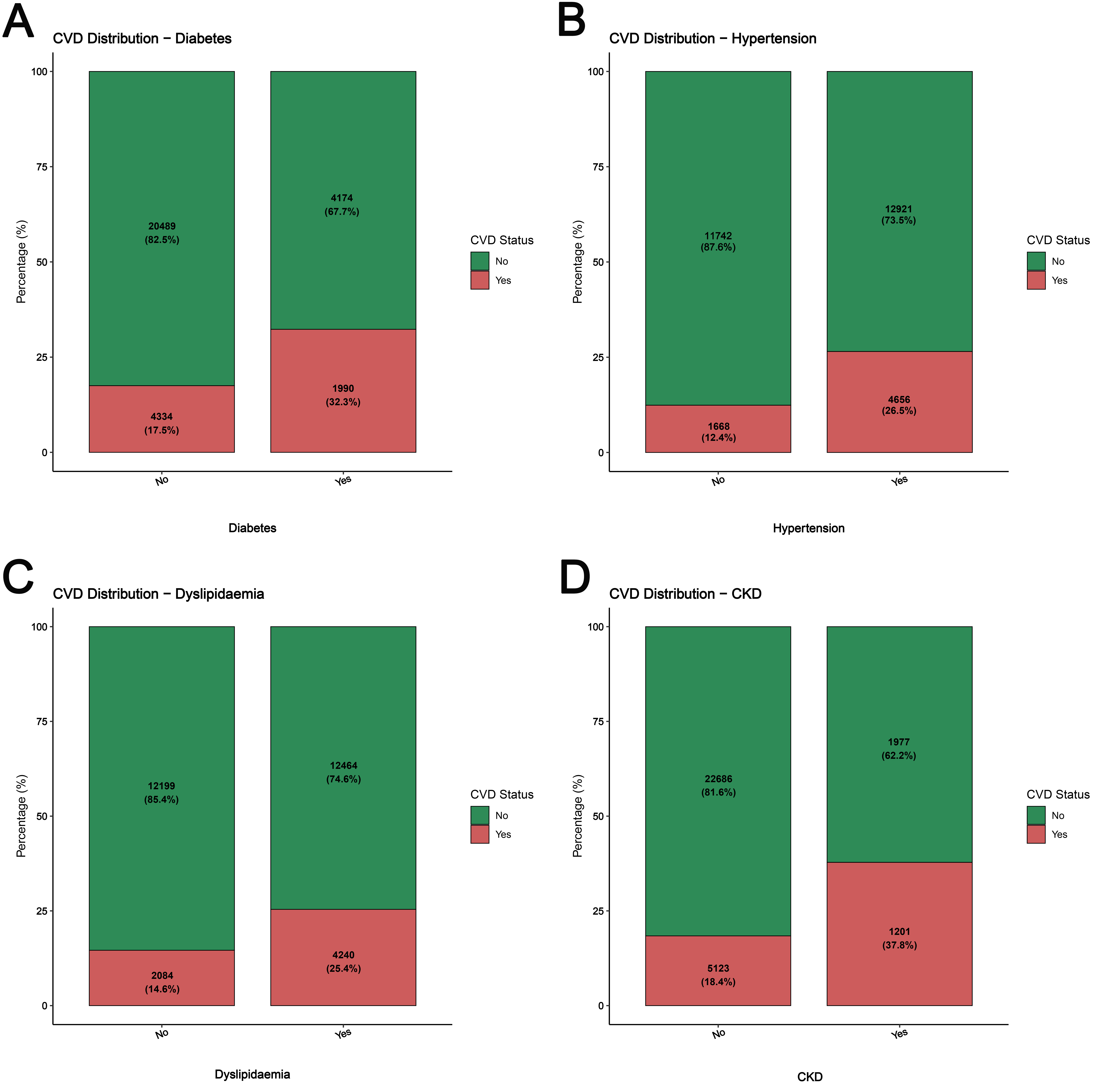

Supplement: Supplementary file 4 — Supplementary Figure S4 Cardiovascular disease in cancer survivors: Case numbers and proportions across clinical subgroups. (A) Diabetes; (B) Hypertension; (C) Dyslipidemia; (D) Chronic kidney disease (CKD). [file Image4.tif]

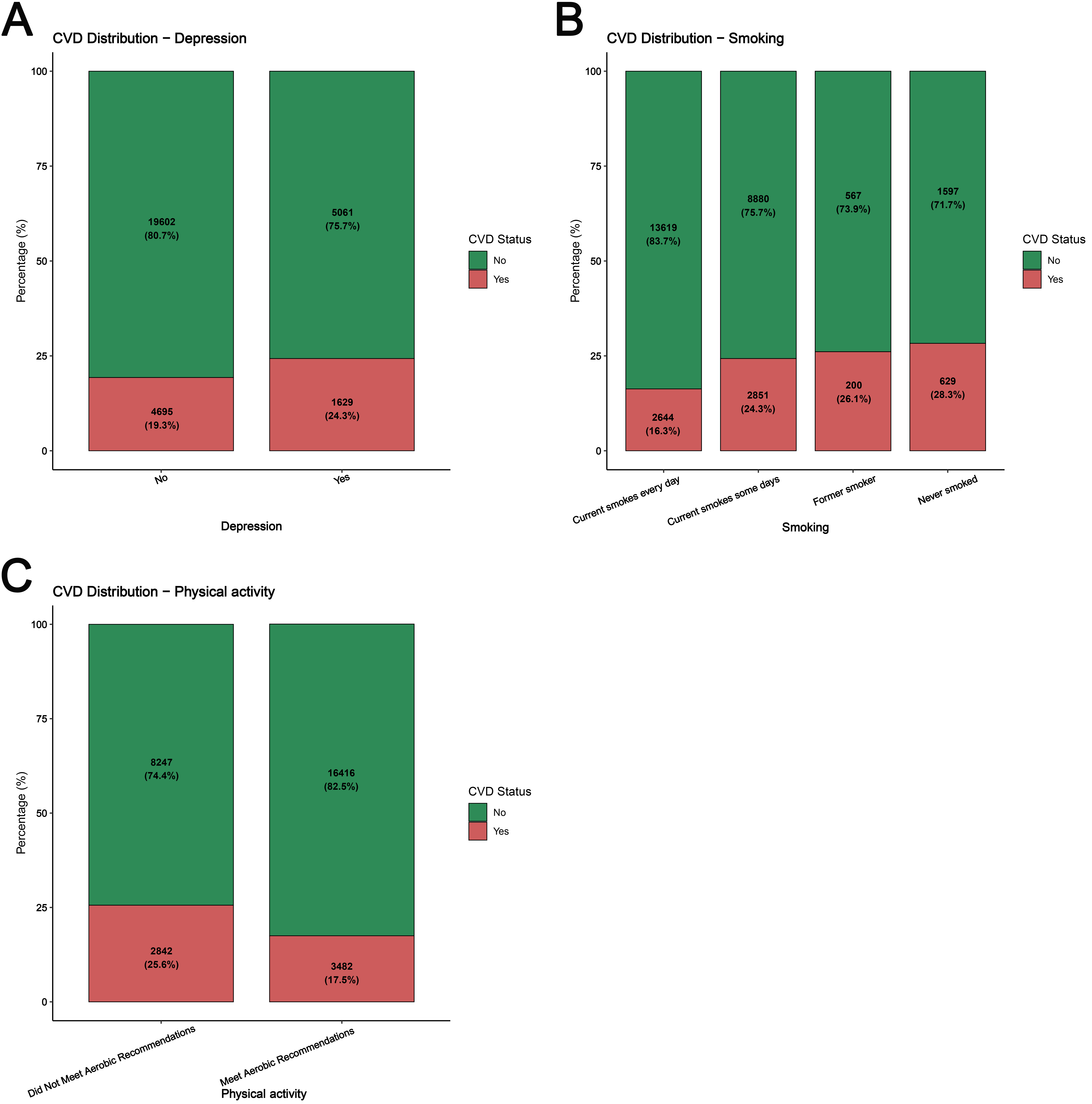

Supplement: Supplementary file 5 — Supplementary Figure S5 Cardiovascular disease in cancer survivors: Case numbers and proportions across clinical subgroups. (A) Depression; (B) Smoking; (C) Physical activity. [file Image5.tif]
