# Supplementary material for: Association between cancer and cardiovascular disease risk: a cross-sectional study of 241,064 individuals
Source: Front Oncol. 2026 Feb 4;16:1734601. doi: 10.3389/fonc.2026.1734601 (PMC12913180; doi:10.3389/fonc.2026.1734601)
Supplement: Supplementary file 7 — Supplementary Table S1 Subgroup analysis of cardiovascular disease risk in cancer survivors versus non-cancer individuals after propensity score matching (PSM). [file Table1.docx]

**Table S1** Subgroup analysis of cardiovascular disease risk in cancer survivors versus non-cancer individuals after propensity score matching (PSM).

| **Demographic characteristics** | **Number (%) (N = 92923)** | **OR (95% CI)** | **P-value** | **P for interaction** |
| --- | --- | --- | --- | --- |
| **Overall** | 92923(100%) | 1.15 (1.11, 1.19) | <0.001 |  |
| **Age** |  |  |  | 0.001 |
| < 71 | 45907(49.4%) | 1.25 (1.18, 1.32) | <0.001 |  |
| ≥ 71 | 47016(50.6%) | 1.11 (1.06, 1.15) | <0.001 |  |
| **Sex** |  |  |  | 0.480 |
| Male | 43267(46.6%) | 1.16 (1.11, 1.22) | <0.001 |  |
| Female | 49656(53.4%) | 1.13 (1.08, 1.19) | <0.001 |  |
| **Marital status** |  |  |  | 0.199 |
| Married | 53720(57.8%) | 1.11 (1.06, 1.17) | <0.001 |  |
| Divorced | 14401(15.5%) | 1.20 (1.10, 1.30) | <0.001 |  |
| Widowed | 16658(17.9%) | 1.20 (1.12, 1.29) | <0.001 |  |
| Single | 8144(8.8%) | 1.21 (1.06, 1.37) | 0.004 |  |
| **Residence** |  |  |  | 0.442 |
| Urban | 79665(85.7%) | 1.15 (1.1, 1.19) | <0.001 |  |
| Rural | 13258(14.3%) | 1.19 (1.09, 1.3) | <0.001 |  |
| **Diabetes** |  |  |  | 0.001 |
| No | 75069(80.8%) | 1.19 (1.14, 1.24) | <0.001 |  |
| Yes | 17854(19.2%) | 1.04 (0.97, 1.11) | 0.252 |  |
| **Hypertension** |  |  |  | <0.001 |
| No | 40641(43.7%) | 1.29 (1.21, 1.38) | <0.001 |  |
| Yes | 52282(56.3%) | 1.10 (1.05, 1.15) | <0.001 |  |
| **Dyslipidaemia** |  |  |  | 0.006 |
| No | 42819(46.1%) | 1.24 (1.17, 1.31) | <0.001 |  |
| Yes | 50104(53.9%) | 1.12 (1.07, 1.17) | <0.001 |  |
| **CKD** |  |  |  | <0.001 |
| No | 83976(90.4%) | 1.17 (1.13, 1.22) | <0.001 |  |
| Yes | 8947(9.6%) | 0.99 (0.90, 1.08) | 0.761 |  |
| **Depression** |  |  |  | 0.707 |
| No | 73207(78.8%) | 1.16 (1.11, 1.20) | <0.001 |  |
| Yes | 19716(21.2%) | 1.14 (1.06, 1.22) | <0.001 |  |
| **Physical activity** |  |  |  | 0.268 |
| Did not meet aerobic recommendations | 32606(35.1%) | 1.12 (1.06, 1.18) | <0.001 |  |
| Meet aerobic recommendations | 60317(64.9%) | 1.17 (1.12, 1.22) | <0.001 |  |

**Abbreviations:** BMI: Body mass index; CI: Confidence interval; CKD: Chronic kidney disease; CVD: Cardiovascular disease; OR: Odds ratio.
